# Sublimation aides and abets co-milling and discoloration involving quinhydrone

**DOI:** 10.3389/fchem.2026.1741180

**Published:** 2026-02-09

**Authors:** Charles Izuchukwu Ezekiel, Leonard R. MacGillivray

**Affiliations:** 1 Department of Chemistry, University of Iowa, Iowa City, IA, United States; 2 Department of Chimie, Université de Sherbrooke, Sherbrooke, QC, Canada

**Keywords:** co-milling, discoloration, dismantling, quinhydrone, solid-state, sublimation

## Abstract

We report an application of co-milling to the binary cocrystal **(BZQ)·(HQ)** or commonly known as quinhydrone. The co-milling is performed with either *trans*-bis(4-pyridyl)ethylene **(4.4′-BPE)** or 4-methoxyaniline **(4-MA)**. In both cases, the dark green color of **(BZQ)·(HQ)** in the sample undergoes discoloration with the co-milling. Sublimation of **BZQ** occurs with dismantling of **(BZQ)·(HQ)** to allow for formation of the targeted cocrystals **(HQ)·(4,4′-BPE)** and **(HQ)·2(4-MA)**.

## 1 Introduction

Quinhydrone - the binary cocrystal **(BZQ)·(HQ)** (where: **BZQ** = *p*-benzoquinone and **HQ** = hydroquinone) - is regarded as the first known cocrystal. The solid was originally reported by Wӧhler in 1844 (Barone et al., 2014; Sakurai, 1965; Wöhler, 1844). **(BZQ)·(HQ)** is deep green in color and forms upon co-grinding of pale-yellow **BZQ** and colorless **HQ**. An X-ray determination of quinhydrone demonstrated the components to assemble by a combination of intermolecular hydrogen bonding and π-π stacking (Sakurai, 1965). Effects of charge transfer have been used to account for the deep green color (Pananusorn et al., 2022; Regeimbal et al., 2003). Quinhydrone has subsequently emerged as a model to evaluate oligomers of biopolymers of melamine and carboxylic equivalents, which have applications in biology, electronics, and related photoelectronic devices (Ariese et al., 2004; Tossell, 2009).

Recent efforts by us have reported an application of co-milling to the orange-red zwitterionic cocrystal (**PDA**)·(**APAP**) (where: **PDA** = 2,4-pyridinedicarboxylic acid, **APAP** = acetaminophen) (Sander et al., 2010). The milling involved co-grinding of (**PDA**)·(**APAP**) with **4,4′-BPE** (where: **4,4′-BPE** = *trans*-1,2-bis(4-pyridyl)ethylene) as solid reagents. Dismantling of (**PDA**)·(**APAP**) afforded the binary cocrystal (**PDA**)·(**4,4′-BPE**) (Ezekiel et al., 2024b). The co-milling involving the orange-red solid resulted in the sample turning colorless. Given that **(BZQ)·(HQ)** is deep green in color, we turned to apply co-milling to **(BZQ)·(HQ)**.

In contrast to (**PDA**)·(**APAP**), **(BZQ)·(HQ)** is regarded as a neutral cocrystal, meaning that each component is devoid of a formal charge (*cf.* Zwitterionic **PDA**). Co-milling is an emerging approach to perform mechanochemical syntheses, being attractive for the design and formation of crystalline phases. Given the shallow landscape of organic solid-state materials, the development of approaches that allow for successful generation of targeted multi-component solids is critical.

**SCHEME 1:**
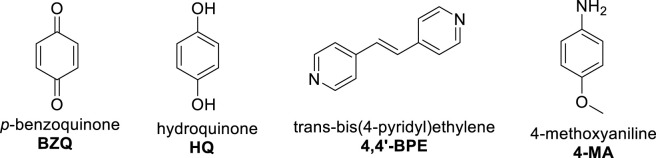
Structures of components used in study.

**SCHEME 2:**
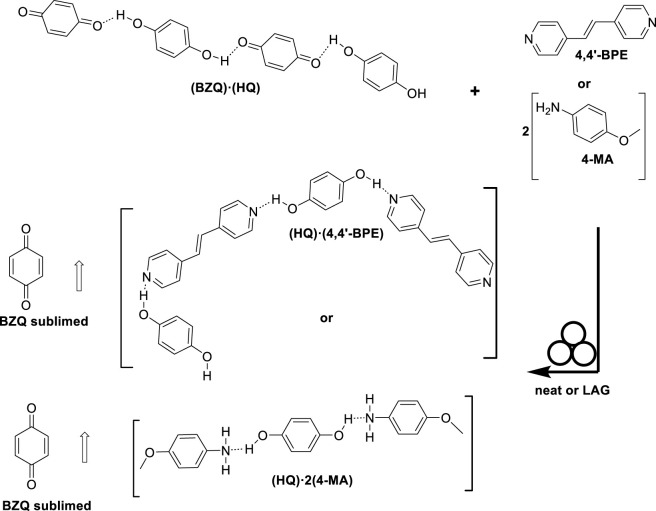
Dismantling of **(BZQ)·(HQ)** through co-milling.

Herein, we report application of co-milling to **(BZQ)·(HQ)**. We show co-milling of **(BZQ)·(HQ)** using either **4,4′-BPE** or **4-MA** (Scheme 1) to result in dismantling of **(BZQ)·(HQ)** through cocrystal exchange reactions that generate known **(HQ)·(4,4′-BPE)** or **(HQ)·2(4-MA)** (Supplementary Figures S1, S2, Supplementary Material) (Ezekiel et al., 2024a; Siva et al., 2020; Weyna et al., 2009). The exchange reactions are accompanied by discolorations wherein the deep green color of each solid sample changes to light beige or dark brown. Importantly, we show the process of sublimation of **BZQ**, which involves physical removal of **BZQ** from the solid sample, to help promote formation and isolation of the targeted co-crystalline solids (Scheme 2). We are unaware of a case wherein sublimation is employed to promote formation and isolation of a cocrystal in a co-milling experiment.

## 2 Methodology

### 2.1 Materials

All reagents and solvents were purchased from commercial sources and generally used as received. **BZQ, HQ, 4,4′-BPE,** and **4-MA** were purchased from Fisher scientific. Ethanol and diethyl ether were purchased from Millipore-Sigma.

### 2.2 Mechanochemistry

Co-millings were performed using a FTS-1000 shaker mill. All experiments were performed either neat or using 10 µL of ethanol in the case of liquid-assisted grinding (LAG) in a stainless steel jar (5 mL) using steel ball bearings (2 × 5 mm) at 20 Hz for a period of up to 60 min. The cocrystal **(BZQ)·(HQ)** used in the dismantlings was formed by milling **BZQ** and **HQ** (1:1 ratio) by LAG with diethyl ether (Sykes et al., 2011) and confirmed by matching calculated and experimental PXRD (Supplementary Figure S3, Supplementary Material). The cocrystal exchange reactions were performed with either **4,4′-BPE** (1:1 ratio) or **4-MA** (1:2 ratio). The calculated PXRD patterns of **(HQ)·(4,4′-BPE)** and **(HQ)·2(4-MA)** match experimental (Supplementary Figures S4, S5, Supplementary Material).

### 2.3 Sublimations

Powder samples of co-milled (**BZQ)·(HQ)** with each of **4,4′-BPE** and **4-MA** were placed in a glass vial connected through vacuum for 72 h.

### 2.4 Powder X-Ray diffraction (PXRD)

Samples for PXRD analyses were ground using a mortar and pestle to generate a uniform powder, which was then deposited on a KS Analytics zero background holder and analyzed with a Bruker D8 Advanced PXRD diffractometer. Data were collected over the range of 5°–40° 2-theta using a 1.5 s step with synchronous rotation of the sample holder.

### 2.5 NMR spectroscopy

Proton nuclear magnetic resonance (^1^H NMR) spectra were recorded at room temperature on a Bruker DRX-400 spectrometer at 400 MHz.

## 3 Results and discussion

Dark green **(BZQ)·(HQ)** is stabilized by a combination of O-H···O hydrogen bonds and charge-transfer between the electron donor (**HQ**) and electron acceptor (**BZQ**). At the molecular level, applications of **(BZQ)·(HQ)** to measure hydrogen ion concentration and in potentiometric titrations have been reported while the cocrystal is a promising cathode material for batteries (Choi, 2019; Curtin et al., 1984; Patil et al., 1984; Patil et al., 1986). **BZQ** itself is used in applications of redox processes (e.g., electron carriers, organic synthesis) (Dandawate et al., 2010). Owing to weak intermolecular interactions in the solid state, **BZQ** readily sublimes as a pure form (Emel et al., 2017). **HQ** experiences applications in pharmaceutical and photographic systems (Ghanbarzadeh et al., 2015; Lin et al., 2005; Nordlund et al., 2006), and the molecule readily oxidizes to form **BZQ** (Brito de Oliveira Moreira et al., 2022).

**FIGURE 1:**
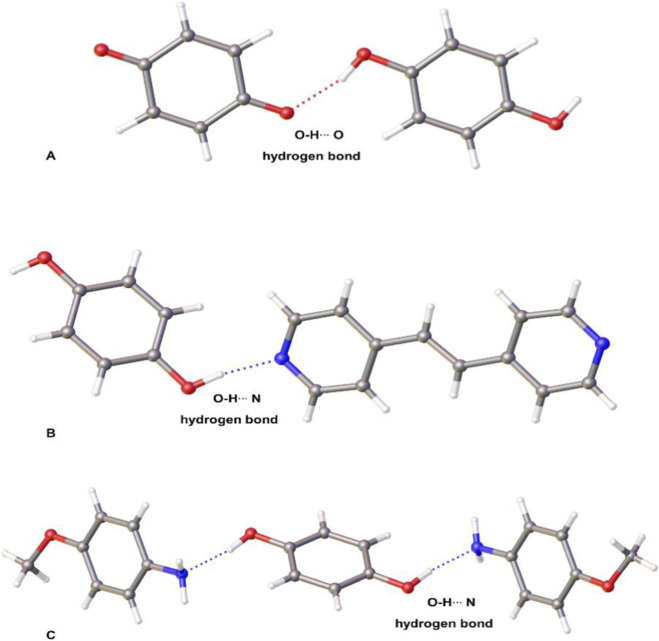
X-ray structures: **(A) (BZQ)·(HQ)** (1245604) (Sakurai, 1965), **(B) (HQ)·(4,4′-BPE)** (730431) (Weyna et al., 2009), and **(C) (HQ)·2(4-MA)** (1583978) (Siva et al., 2020) (CCDC reference numbers parenthesis).

**HQ** is reported to form a total of 110 binary cocrystals (Cambridge Structural Database (CSD) version 5.46 November 2024). **BZQ** forms 47 binary cocrystals. An analysis of the CSD data shows **HQ** to form cocrystals with N-atom hydrogen-bond-acceptors (94 structures total). The O-H···N hydrogen bond is a resaonably reliable supramolecular synthon in synthesis of multicomponent crystals (Khan et al., 2009). Given our report to develop cocrystals of bipyridines through co-milling (Ezekiel et al., 2024b), we hypothesized **(BZQ)·(HQ)** could be dismantled by co-milling with the N-atom hydrogen-bond acceptors **4,4′-BPE** (Weyna et al., 2009) and **4-MA** (Siva et al., 2020) (Figure 1). The acceptors form cocrystals **(HQ)·(4,4′-BPE)** (light yellow) and **(HQ)·2(4-MA)** (black) from solution. The co-millings were expected to result in discolorization of dark green **(BZQ)·(HQ**) to afford samples based on colors of the targeted product cocrystals. The CSD shows **HQ** to form binary cocrystals of linear chains and a discrete aggregate with **4,4′-BPE** and **4-MA**, respectively (Figures 1B,C). The co-millings were expected to involve breakage of the O-H···O hydrogen bonds (2 total) of **(BZQ)·(HQ)** (Table 1) along with formation of O-H···N_pyr_ hydrogen bonds (2 total) for **(HQ)·(4,4′-BPE)** and O-H···N_amino_ (2 total) hydrogen bonds for **(HQ)·2(4-MA)** (Table 1). Solid **BZQ**, which does not participate in the formation of appreciably strong hydrogen bonds, was expected to form as side product.

**TABLE 1:** Hydrogen bonds in co-milling of **(BZQ)·(HQ)**.

(BZQ)·(HQ) hydrogen bonds broken (total)	Hydrogen bonds formed (total)	
	(HQ)·(4,4′-BPE) (chains)	(HQ)·2 (4-MA) (aggregate)
O-H‧‧‧O (2)	O-H‧‧‧N_pyr_ (2)	O-H‧‧‧N_amino_ (2)

**FIGURE 2:**
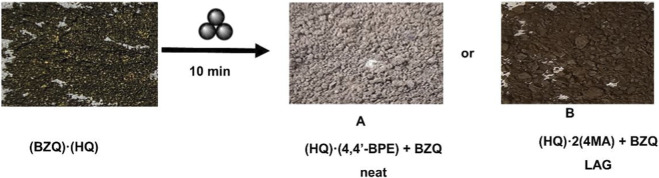
Photographs of solids (placed on filter paper) from co-millings of **(BZQ)·(HQ)**.

**FIGURE 3:**
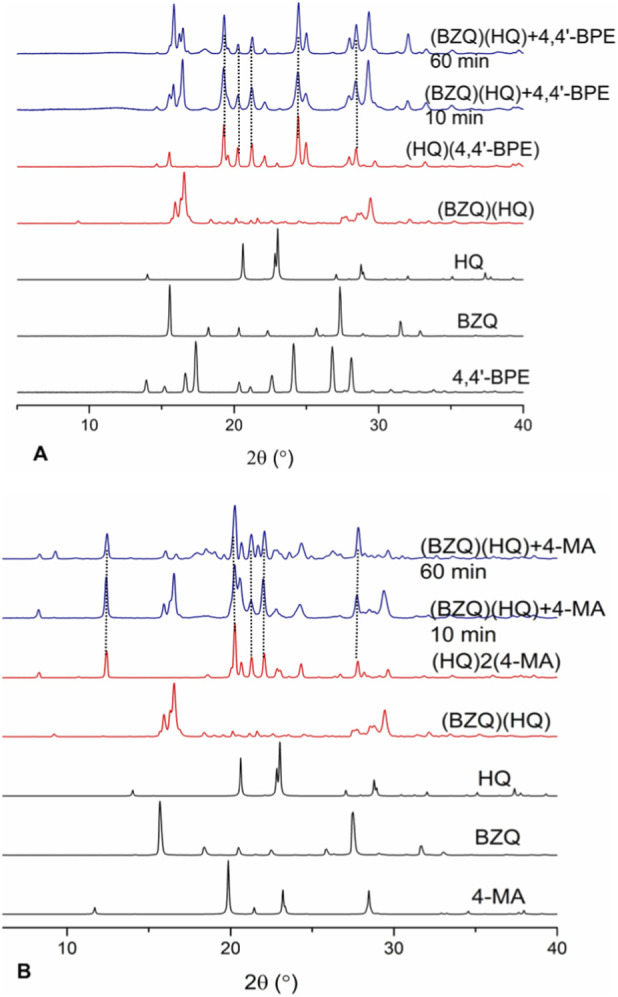
PXRD diffractograms co-millings of **(BZQ)·(HQ)**: **(A) 4,4′-BPE** (neat) and **(B) 4-MA** (LAG).

When **(BZQ)·(HQ)** was subjected to co-milling with **4,4′-BPE** by neat grinding (10 min) (Table 2), the dark green color changed to light beige (Figure 2A). Five prominent peaks emerged in the PXRD diffractogram (2θ = 19.3°, 20.2°, 21.3°, 24.4, 28.4°) (Figure 3A). The peaks were consistent with the formation of **(HQ)·(4,4′-BPE)**. The cocrystal **(HQ)·(4,4′-BPE)** is reported as light-yellow. Peaks attributed to **(BZQ)·(HQ)** (2θ = 15.8°, 16.5°, 29.4°), **BZQ** (2θ = 15.5°), **4,4′-BPE** (2θ = 28.1°) were also present. A longer co-milling time (60 min) did not result in an appreciable change in color of the solid sample.

**TABLE 2:** Dismantling of **(BZQ)·(HQ)** by co-milling.

Coformer	Co-millings		Final color
	LAG	Neat	
**4,4′-BPE**	No (ethanol)	Yes	Beige
**4-MA**	Yes (ethanol)	Yes	Dark brown

When **(BZQ)·(HQ)** was subjected to co-milling with **4-MA** by LAG (10 min, ethanol), the dark green color changed to dark brown (Figure 2B). The PXRD diffractogram showed the emergence of five prominent peaks (2θ = 12.5°, 20.2°, 21.3° 22.1°, 27.8°). The peaks were consistent with the formation of **(HQ)·2(4-MA)**. The color of **(HQ)·2(4-MA)** is reported as black. Four peaks of reduced intensities (2θ = 15.8, 16.5°, 29.4°) attributed to **(BZQ)·(HQ)** were also present (Figure 3B). We note that peaks attributed to neither **BZQ** nor **4-MA** were present. A longer milling time (60 min) did not result in a change in color. The generation of **(HQ)·2(4-MA)** was also realized by neat grinding.

**FIGURE 4:**
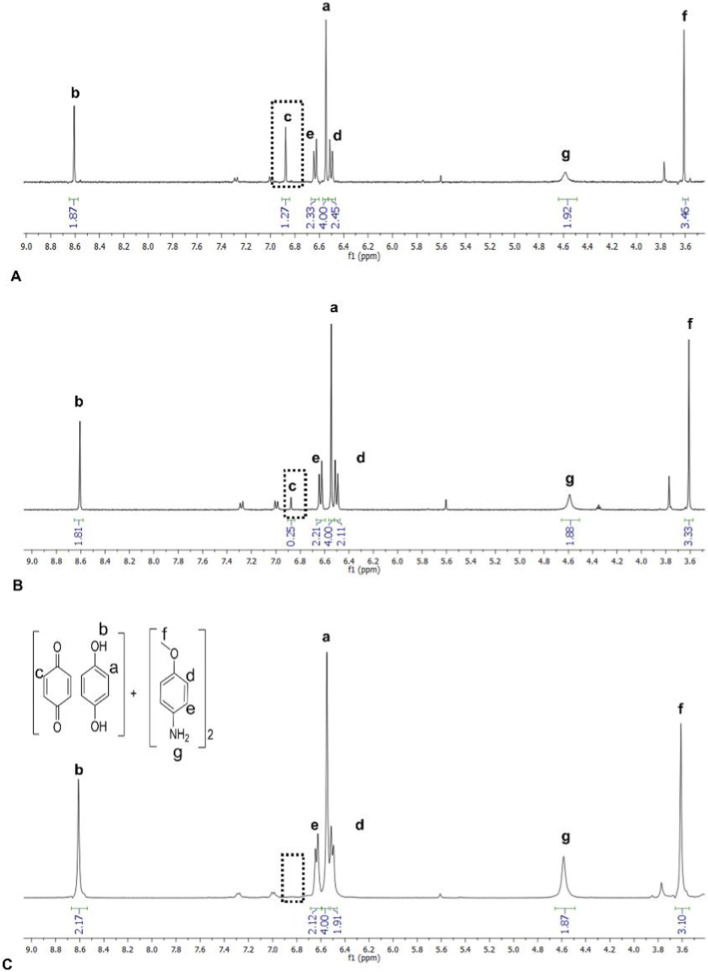
^1^H NMR spectra after co-milling **(BZQ)·(HQ)** with **4-MA**: **(A)** 10 min (LAG EtOH), **(B)** 60 min (LAG EtOH), and **(C)** 72 h (sublimation). Singlet peak inside the box represents **BZQ**.

**FIGURE 5:**
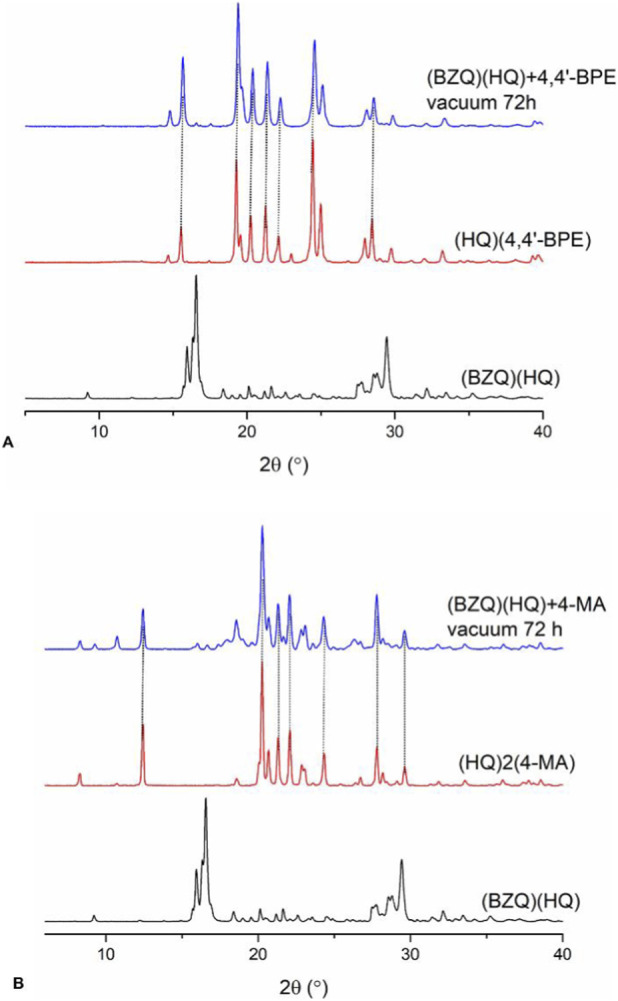
PXRD diffractograms following co-milling of **(BZQ)·(HQ)** under vacuum: **(A) 4,4′-BPE** and **(B) 4-MA**.

While the PXRD data showed **(BZQ)·(HQ)** to be dismantled in each co-milling, the amount of **BZQ** that remained in each sample varied. From ^1^H NMR data, over half **BZQ** (0.75 equivalent) remained upon co-milling with **4,4′-BPE** (10 min) (Supplementary Figure S6, Supplementary Material). Significantly less **BZQ** (0.32 equivalent) remained following co-milling with **4-MA** (Figure 4A). For the longer co-milling time (60 min), **BZQ** that remained was either relatively unchanged or significantly less for **4,4′-BPE** (0.75 equivalent) and **4-MA** (0.06 equivalent), respectively (Figure 4B). When each co-milled sample was also subjected to moderate vacuum (72 h), **BZQ** was completely removed in each case (Figure 4C), (Supplementary Figure S6, Supplementary Material). The resulting PXRD diffractograms were consistent with either **(HQ)·(4,4′-BPE)** or **(HQ)·2(4-MA)** being present (Figure 5) (Supplementary Table S2, Supplementary Material).

The losses of **BZQ** in the co-millings can be attributed to effects of sublimation (Acree and Chickos, 2010; Reid et al., 1959; Červinka and Fulem, 2017). **BZQ** readily sublimes at room temperature, which is reflective of weak intermolecular forces in pure **BZQ** (Lin et al., 2021). In previous work, Groeneman employed sublimation to remove a halogen-bond-donor coformer to isolate a cyclobutane photoproduct (Grobelny et al., 2017). Mei also used sublimation to remove halogen-bond-donor coformers from a photodimer of vitamin K3 (Zhu et al., 2016). Our group has recently attributed a decrease of a hydrogen-bond-donor coformer in a solid-state photoreaction to sublimation (Oburn et al., 2019). For the current work, sublimation of the hydrogen-bond-acceptor coformer **BZQ** can be regarded as a means to aide the generation and isolate a cocrystal as a product of a co-milling (Carstens et al., 2020). We are unaware of a case wherein sublimation in co-milling aides and abets the generation of a cocrystal. Similar to (**PDA**)·(**APAP**) (Ezekiel et al., 2024b), the cocrystal exchange can be explained on the basis of melting point. The binary cocrystals **(HQ)·(4,4′-BPE)** (224 °C–225 °C) (Quentin and MacGillivray, 2020), and **(HQ)·2(4-MA)** (191 °C) (Siva et al., 2020) melt at higher temperatures *versus* **(BZQ)·(HQ)** (167 °C–172 °C) (Curtin et al., 1984).

## 4 Conclusion

In our report, we demonstrated sublimation to support co-millings involving **(BZQ)·(HQ)**, with the co-millings resulting in discolorations of the solid samples. We are currently expanding applications of co-milling to **(BZQ)·(HQ)**, as well as identifying additional components that sublime and can serve as candidates in co-crystal generation. Understanding mechanisms responsible for dismantling cocrystals with the use of sublimation can be expected to influence conformer selections in the design and manufacturing of multicomponent crystals.

## Data Availability

The original contributions presented in the study are included in the article/Supplementary Material; further inquires can be directed to the corresponding author.
