# Supplementary material for: Sublimation aides and abets co-milling and discoloration involving quinhydrone
Source: Front Chem. 2026 Feb 9;14:1741180. doi: 10.3389/fchem.2026.1741180 (PMC12925636; doi:10.3389/fchem.2026.1741180)
Supplement: Supplementary file 1 [file DataSheet1.pdf]

Supplementary Material

Sublimation Aides and Abets Co-millings and Discolorations Involving Quinhydrone

Charles Izuchukwu Ezekiel,[1] and Leonard R. MacGillivray[*,1,2]

[1] Department of Chemistry, University of Iowa, Iowa City, IA 52242, USA

[2] Département de Chimie, Université de Sherbrooke, Sherbrooke, QC, J1K 2R1, Canada

E-mail: leonard-macgillivray@usherbrooke.ca

Supplementary Material

1. Experimental information

2. Table S1. Co-milling parameters **of (BZQ)·(HQ)** with **4,4'-BPE** and **4-MA**.

3. Figure S1. Reported crystal structure of **(HQ)·(4,4'-BPE)**.

4. Figure S2. Reported crystal structure of **(HQ)·2(4-MA)**.

5. Figure S3. Experimental and simulated PXRD patterns of **(BZQ)·(HQ)**

6. Figure S4. Experimental and simulated PXRD patterns of **(HQ)·(4,4'-BPE)**

7. Figure S5. Experimental and simulated PXRD patterns of **(HQ)·2(4-MA)**

8. Figure S6. ^{1}H NMR spectra of **(BZQ)·(HQ)+4,4'-BPE)**

9. Table S2. Parameters and photographs of solid samples in co-millings.

Experimental Information

Mechanochemistry. Co-milling was performed using a FTS-1000 shaker mill. All co-milling experiments were performed either neat or using 10 µL of ethanol in the case of liquid-assisted grinding (LAG) in a stainless steel jar (5 mL) using steel ball bearings (2 x 5 mm) at 20 Hz for a period of up to 60 minutes. The cocrystal **(BZQ)·(HQ)** used in a dismantling was formed by milling **BZQ** and **HQ** (1:1 ratio) by LAG. The cocrystal exchange reactions were performed with either **4,4'-BPE** (1:1 ratio) or **4-MA** (1:2 ratio). The details of the experiment is reported in Table S1.

Powder X-ray diffraction. PXRD data were collected on a Bruker D8 Advance X-ray diffractometer using CuKα1 radiation (λ = 1.5418 Å) in the range 5–40° (scan type: coupled TwoTheta/Theta; scan mode: continuous PSD fast; step size: 0.019°) (40 kV and 30 mA).

Sublimation. Powder samples of co-milled (**BZQ)·(HQ)** with each of **4,4'-BPE** and **4-MA** (5 mg) were placed in a glass vial under vacuum for 24 h or 72 h.

NMR spectroscopy. Proton nuclear magnetic resonance (^{1}H NMR) spectra were recorded at room temperature on a Bruker® DRX-400 spectrometer at 400 MHz (instrument parameters: field strength: 9.2 T; RF-Console: DRX 3-channel; magnet: shielded superconducting; probe: nature, 5.0 mm BBO-^{1}H type, double-resonance; temperature range: 100-180 °C).

Table S1. Co-milling parameters of **(BZQ)·(HQ)** with **4,4'-BPE** and **4-MA**.

Time (min)	(BZQ)·(HQ) (mg)	4,4'-BPE (mg)
10	25.7	21.5
60	24.1	20.2

Time (min)	(BZQ)·(HQ) (mg)	4-MA (mg)
10	23.3	26.4
60	22.9	25.9

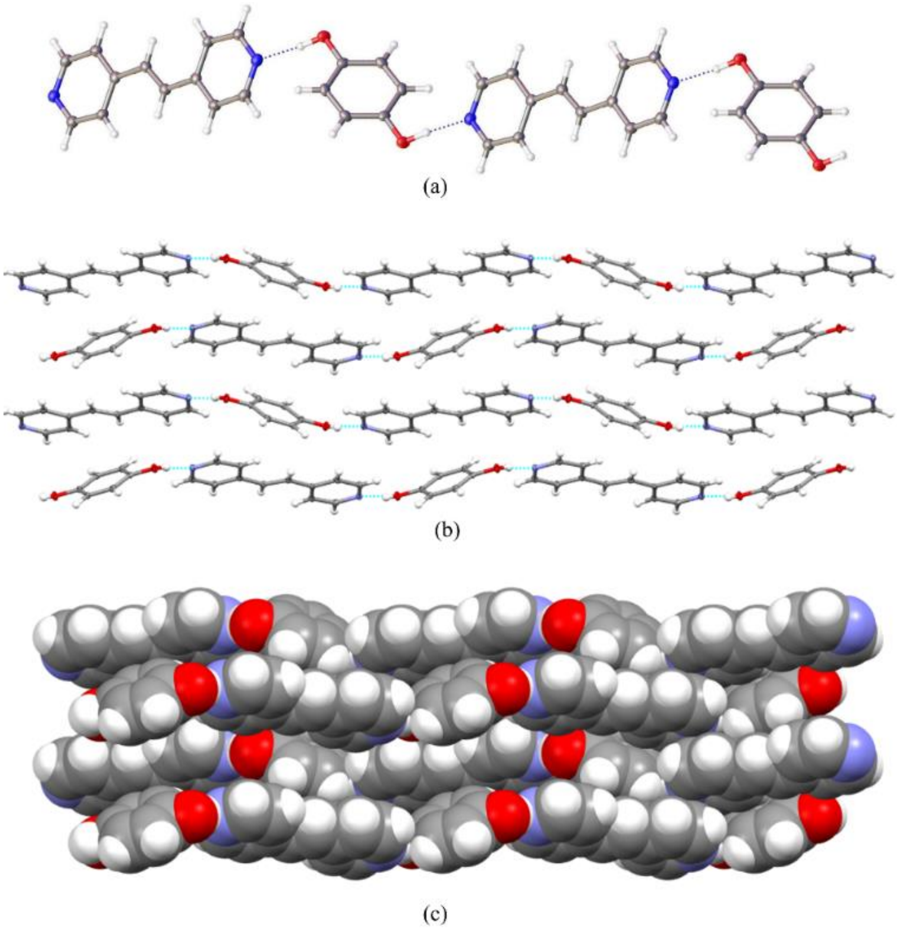

Figure S1. Reported crystal structure of **(HQ)·(4,4'-BPE):** (A) linear chain, (B) extended planar sheet**,** and (C) planar sheet (space-filling). Adapted from CCDC ref no 730431(Weyna et al., 2009).

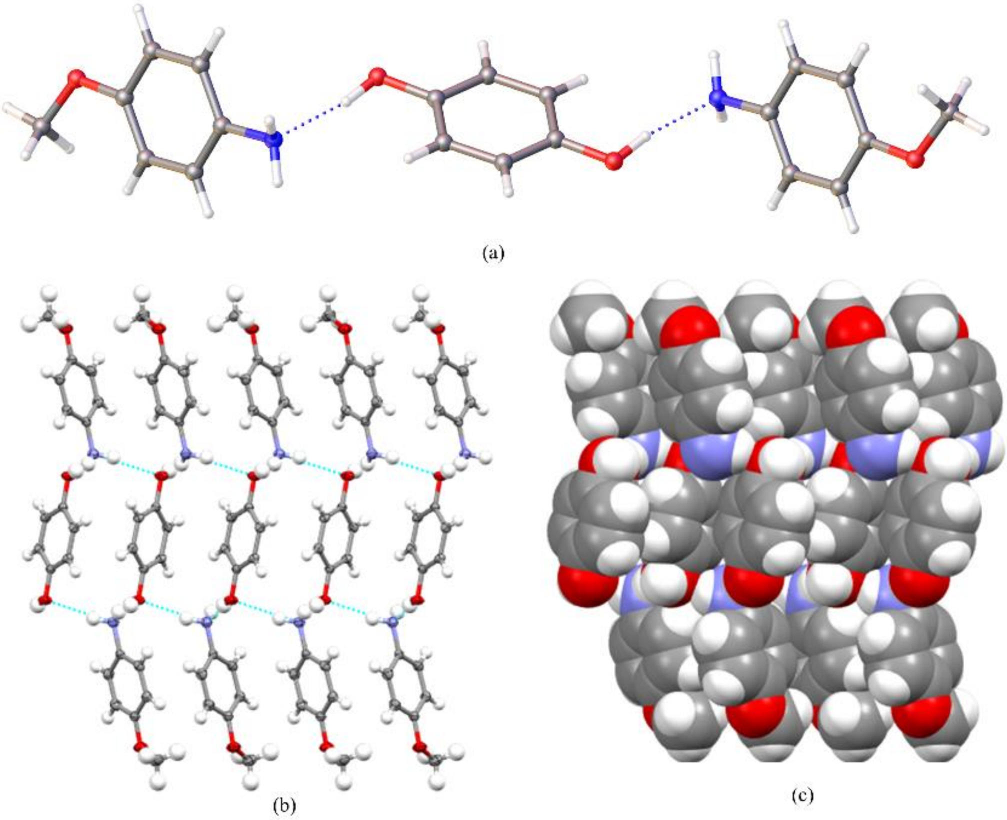

Figure S2. Reported crystal structure of **(HQ)·2(4-MA):** (A) linear chain, (B) extended planar sheet**,** and (C) planar sheet (space-filling). Adapted from CCDC ref no 1583978 (Siva et al., 2020).

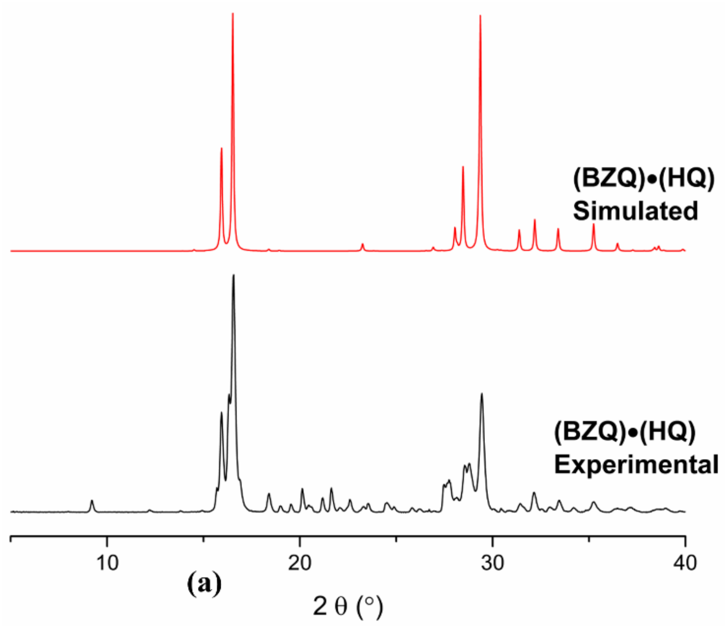

Figure S3. Experimental and simulated PXRD patterns of **(BZQ)·(HQ).**

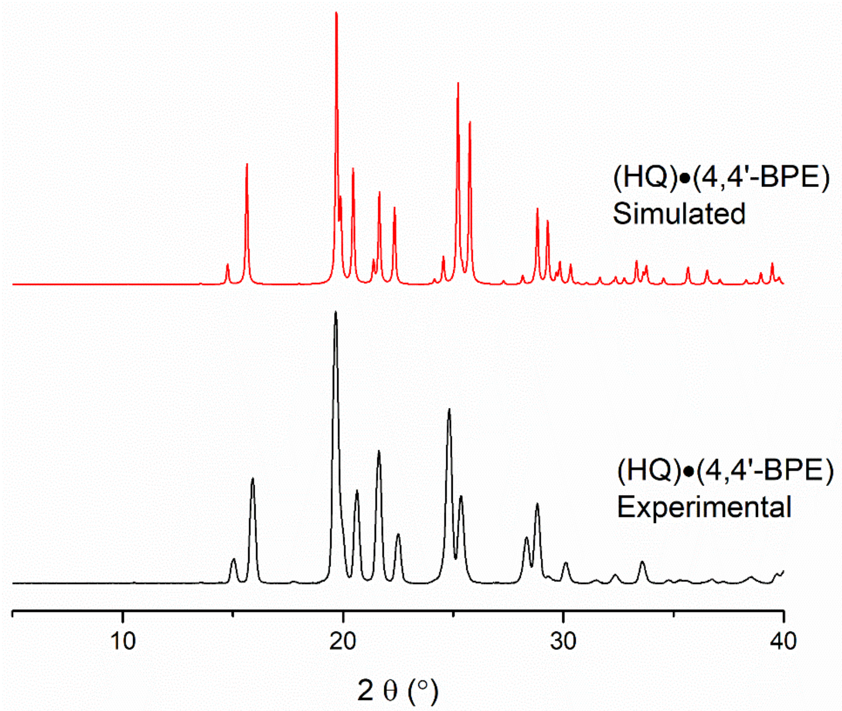

Figure S4. Experimental and simulated PXRD patterns of **(HQ)·(4,4'-BPE).**

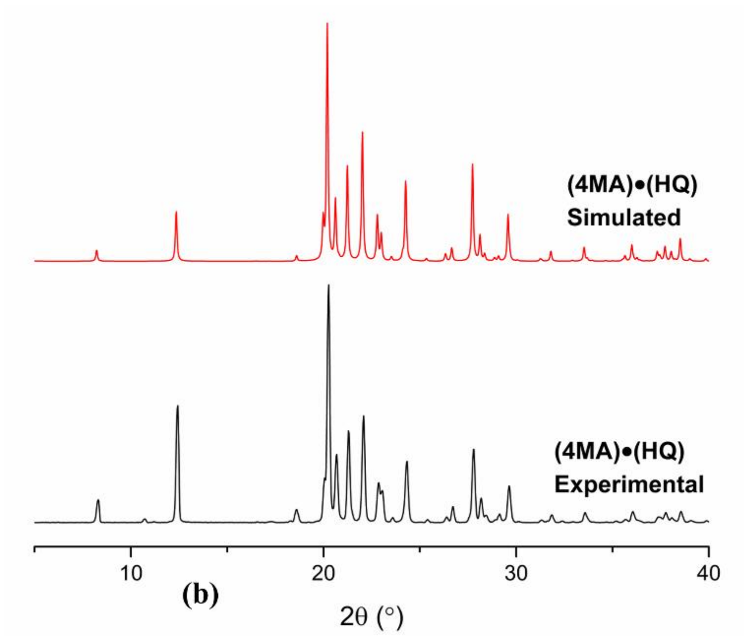

Figure S5. Experimental and simulated PXRD patterns of **(HQ)·2(4-MA).**

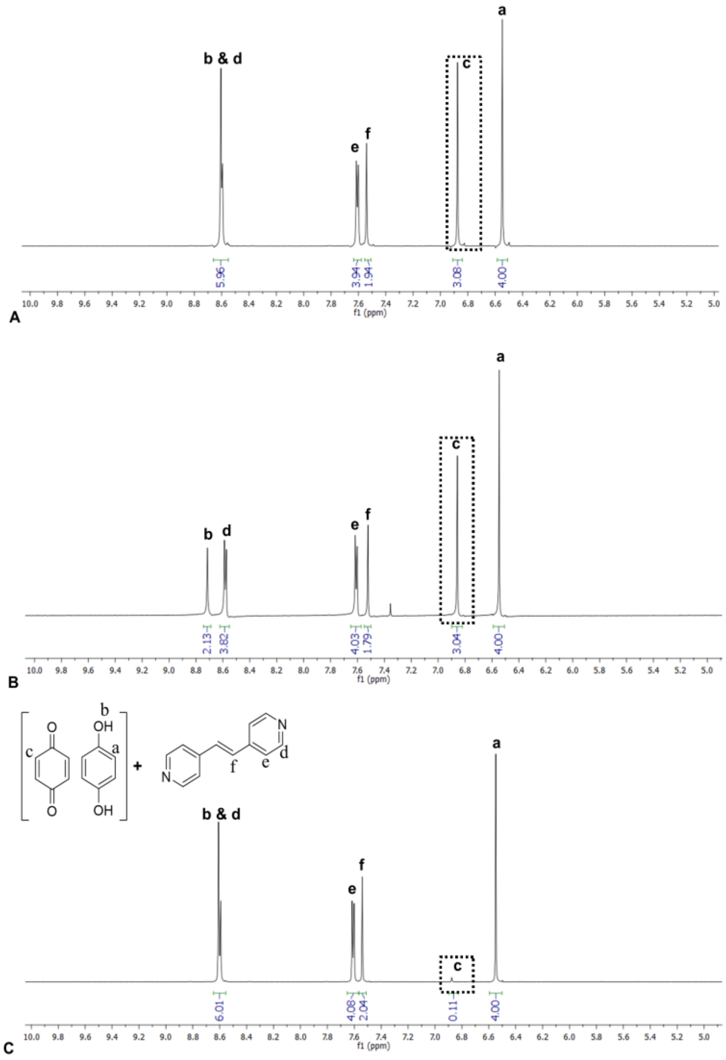

Figure S6. ^{1}H NMR spectra of **(BZQ)·(HQ)** after co-milling (neat grinding) with **4,4'-BPE**: (A) 10 min, (B) 60 min, and (C) 72 h (sublimation). Singlet peak inside the box represents **BZQ**.

Table S2. Parameters and photographs of solid samples in co-millings.

Components	Condition	Time	Color
**(BZQ)·(HQ)**	LAG	30 min	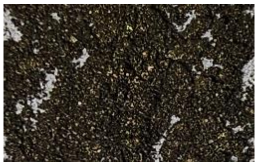
**(BZQ)·(HQ)+4,4'-BPE**	Neat	10 min	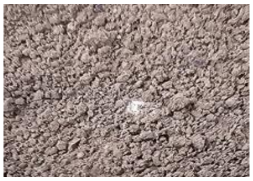
**(BZQ)·(HQ)+4-MA**	LAG	10 min	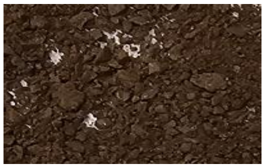
**(HQ)·2(4-MA)**	LAG	30 min	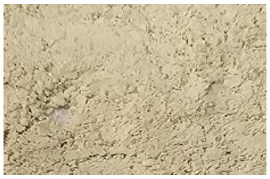
**(HQ)·(4,4'-BPE)**	LAG	30 min	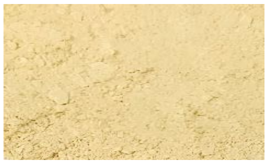
**(BZQ)·(HQ)+4,4'-BPE**	Neat + vacuum	72 h	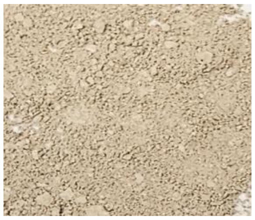

**(BZQ)·(HQ)+4-MA**	LAG + vacuum	72 h	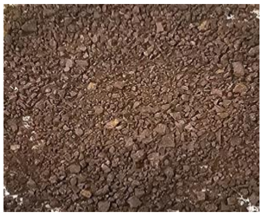

References

Siva, V., Shameem, A., Murugan, A., Athimoolam, S., Suresh, M., and Bahadur, S. A. (2020). Investigation on structural, optical, dielectric, mechanical and thermal properties of 4-methoxyaniline hydroquinone cocrystal: A promising optical power limiting material. *J. Mol. Struct.* 1205, 127619. doi: 10.1016/j.molstruc.2019.127619

Weyna, D. R., Shattock, T., Vishweshwar, P., and Zaworotko, M. J. (2009). Synthesis and Structural Characterization of Cocrystals and Pharmaceutical Cocrystals : Mechanochemistry vs Slow Evaporation from Solution & DESIGN 2009. *Cryst. Growth Des.* 9, 18–25.
